# Synthesis of 6-(2-Methoxynaphthyl)-2,3-dihydro-1,2,4-triazine-3-thione as a New Reagent for Spectrophotometric Determination of Copper

**DOI:** 10.1155/2014/260179

**Published:** 2014-02-04

**Authors:** Maliheh Barazandeh Tehrani, Hutan Ghanbari, Effat Souri, Fazel Shamsa, Mohsen Amini

**Affiliations:** Department of Medicinal Chemistry, Faculty of Pharmacy and Pharmaceutical Science Research Center, Tehran University of Medical Sciences, Tehran 1417614411, Iran

## Abstract

A simple, sensitive, accurate, and green spectrophotometric method for the determination of Cu(II) using newly synthesized reagent, 6-(2-methoxynaphthyl)-2,3-dihydro-1,2,4-triazine-3-thione (MNDTT), has been developed. MNDTT was synthesized based on the acylation of methoxy naphthalene and reaction of the product with amyl nitrite, which upon reaction with thiosemicarbazide yielded 6-(2-meyhoxynaphthyl)-2,3-dihydro-1,2,4-triazine-3-thione. MNDTT produces a dark red complex with copper in methanol according to the 1 : 2 stoichiometry. Beer's law was obeyed over the concentration range of 2.5–20 *µ*g/mL with *r* ^2^ = 0.992. The limit of detection and limit of quantification were 0.33 and 1.10 *µ*g/mL, respectively. Within-day and between-day precision values were less than 3.68%. Finally, the method has been applied to a dental alloy (110-plus) successfully and the results were compared with atomic absorption method. The results showed that there was no significant difference between the two methods (*P* > 0.05).

## 1. Introduction

Copper is an essential micronutrient for many vital processes. Copper is present in a large number of enzymes, which are involved in electron transfer, activation of oxygen, and other small molecules as well as superoxide dismutation [1, 2]. It serves as an essential cofactor for a variety of proteins involved in neurotransmitter synthesis as well as in neuroprotection via the Cu/Zn superoxide dismutase. Copper in excess is toxic while Cu deficiency can lead to serious disease [3–5]. Copper ions have also showed antimicrobial activity against a wide range of microorganisms [6–8].

The determination of trace amounts of copper, because of its importance in health, medical, and industrial processes, is of great interest to analytical chemists. There are many reported methods for the determination of copper. The most common techniques are ICP-MS [9, 10], atomic absorption [11, 12], capillary electrophoresis [13], and UV/VIS spectrophotometry [14–16]. Most of these methods are time consuming or require expensive and complicated instruments. In addition, some methods use organic solvents such as chloroform or acetonitrile which are for the issue of environmental concern or worldwide storage crisis.

Among these techniques, visible absorption spectrophotometry represents the most convenient technique because of the availability of the instrumentation, simplicity, speed, precision, accuracy, and low cost. As it was conducted in our previous studies, 6-naphthyl and anthracenyl substituted, 1,2,4-triazine-3-thione form colored complexes with Cu(II) in basic media [17–20]. In this investigation the synthesis of 6-(2-methoxynaphthyl)-2,3-dihydro-1,2,4-triazine-3-thione has been synthesized as a chromogenic reagent for the determination of Cu(II) and validation of the developed method is reported.

## 2. Experimental

### 2.1. Apparatus

All spectra recordings and absorbance measurements were carried out on a Shimadzu, 160A UV/VIS spectrophotometer. Atomic absorption (AA Varian 220) was used for Cu determination in real samples. NMR spectra were recorded on a Bruker FT-500 Spectrometer (Bruker, Rheinstetten, Germany) with tetramethyl silane (TMS) as internal standard. Infrared spectra were obtained using a Perkin-Elmer Model 781 spectrograph. Mass spectra were taken using a Finnigan TSQ Spectrometer at 70 eV.

### 2.2. Reagents

2-Methoxynaphthalene, thiosemicarbazide, amylnitrite, aluminium chloride, and acetyl chloride were used for ligand synthesis and were purchased from Fluka (Switzerland) or Merck Chemical Companies. Solvents (acetone, acetonitrile, ethyl alcohol, and methanol) were of HPLC grade and prepared from Merck (Germany).

*Copper Nitrate Solution*. A stock standard solution of 1 mg/mL Cu(II) was prepared by dissolving 0.5 g pure elemental copper in hot concentrated HNO_3_, cooling, and adjusting the volume to 500 mL by addition of the distilled water. Working solutions were prepared by proper dilution by ethyl alcohol.

**Figure 1:**
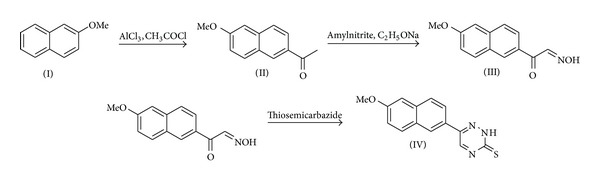
Synthesis of 6-(2-methoxynaphthyl)-2,3-dihydro-1,2,4-triazine-3-thione (MNDTT).

*Synthesis of the Chromogenic Reagent, MNDTT*. The reagent 6-(2-methoxynaphthyl)-2,3-dihydro-1,2,4-triazine-3-thione (MNDTT) was synthesized according to Figure 1.

*Synthesis of 2-Methoxy Acetyl Naphthalene (II)*. Acetyl chloride (0.4 g) and AlCl_3_ (0.67 g) were mixed in a mortar in dry condition and then 2-methoxynaphthalen (I) (0.5 g) was added and mixed in oxygen protected condition. After 45 min cool water was added and the solution washed with ether. The product was extracted with ether and washed with hydrochloric acid and dried. The product was re-crystallized from ethanol-water to yield 0.454 g (72%), Mp. 108–110°C. ^13^C NMR (125 MHz, CDCl_3_) was 32.68 (CH_3_CO), 55.36 (OCH_3_), 112.71, 123.58, 124.05, 128.65, 128.13, 128.78, 130.37, 131.44, 133.31, 153.91, and 205.22.

*Synthesis of 6-(2-Methoxynaphthyl)-2,3-dihydro-1,2,4-triazine-3-thione (MNDTT)(IV)*. The resultant dried precipitate (II) was then refluxed with amyl nitrite in ethanol in presence of sodium ethoxide for 48 h in dry condition. The product (III), 2-methoxynaphthylglyoxal aldoxime, was then extracted with diethyl ether, dried, and crystallized from water-ethanol. The reaction product (III) and thiosemicarbazide were refluxed for 3 h in dilute hydrochloric acid. The progress of the reaction was monitored by TLC using a mixture of chloroform and methanol as a mobile phase. The reaction mixture was then cooled and filtered off. The precipitate was washed with ether and crystallized from DMSO-H_2_O. Compound (IV) was obtained as yellowish brown powder in 58% yield. ^13^C NMR (125 MHz, DMSO) was 57.04 (OCH_3_), 114.25, 123.56, 124.45, 128.03, 128.56, 131.21, 131.26, 132.48, 154.85, 158.53, 173.76, and 188.74. ^1^H NMR (500 MHz, DMSO) was 3.87(s, 3H, CH_3_), 7.38–7.56 (m, 3H), 7.57–7.62 (m, 2H), 810 (s, 1H), and 833 (bs, 1H, NH). Mass *m/z* (%) was 269 (7), 241 (5), 239 (20), 165 (10), 153 (20), 151 (100), and 138 (4).

*General Procedure for Determination of Cu(II)*. In a series of 5 mL volumetric flask, 1 mL (2.5–20 *μ*g/mL) of Cu(II) and 2 mL of MNDTT (0.002 M) were taken and the volume was adjusted to 5 mL with methanol. The absorbance of the solutions was recorded against reagent blank at 475 nm in a 1 cm quartz cell.

*Analytical Application*. 40 mg of an amalgam (110-plus) containing Ag(I) (45%), Sn(II) (30%), and Cu(II)(25%) was taken in a 50 mL beaker and 10 mL HNO_3_ : HCl (1 : 1, v/v) solution was added. The mixture was heated at 120°C for 30 min until the dissolution is completed and the resulting solution reaches minimum volume. The solution cooled and transferred to a 100 mL volumetric flask and diluted to mark with double distilled water. The copper content of the sample was analyzed according to the proposed method.

## 3. Results and Discussion 

The reagent 6-(2-methoxynaphthyl)-2,3-dihydro-1,2,4-triazine-3-thione (MNDTT) was synthesized, due to our recent studies for preparing more sensitive chromogenic reagents, for determination of trace amount of some cations such as Cu(II), Ni(II), and Hg(II). To achieve more sensitivity, complexing moiety, 1,2,4-triazine-3-thione ring, was left intact and the chromogenic part was changed. MNDTT forms a brownish-red complex with Cu(II) in methanol.

Different solvent systems (acetonitrile, water, 0.1 M NaOH, chloroform, ethanol, and methanol) were examined to find out more suitable solubility, better absorption spectra, and greener solvent. The results showed that the reagent, MNDTT, was not soluble in chloroform and water. Both the reagent and Cu-MNDTT complex were soluble in 0.1 M NaOH, acetonitrile, ethanol, and methanol. The absorbance value was very low in basic media. Acetonitrile not only had lower sensitivity but also was not considered as an environmental and health friendly solvent [25, 26]. Eventually, comparison of the absorption value of Cu-MNDTT complex in methanolic and ethanolic media showed that the most suitable solvent for both reagent and complex was methanol (Table 1). Therefore, methanol which had greater absorbance value and was also considered as one of the greener solvents was selected for subsequent experiments.

**Figure 2:**
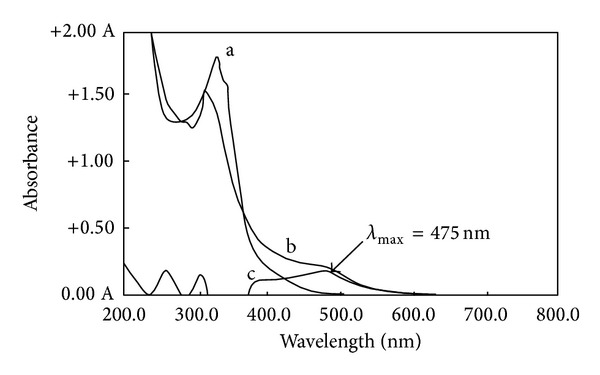
UV-VIS absorption spectra of (a) MNDTT, (b) Cu-MNDTT complex, and (c) Cu-MNDTT complex against reagent blank (*λ*_max⁡_ = 475 nm).

**Figure 3:**
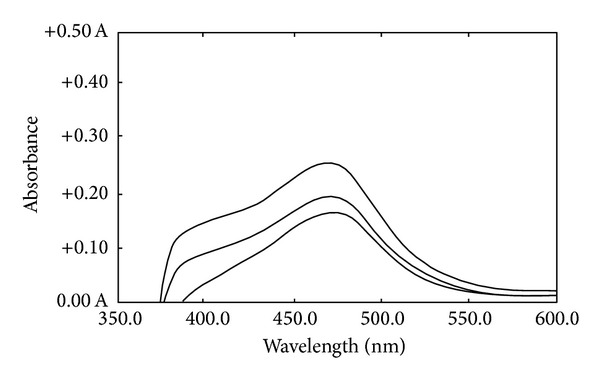
Absorption spectra of Cu-MNDTT complex in three concentrations, 15, 17.5, and 20 *μ*g/mL.

*Absorption Spectra*. 6-(2-Methoxynaphthyl)-2,3-dihydro-1,2,4-triazine-3-thion, which is synthesized as a new reagent, reacts with Cu(II) forming a red-colored complex in methanol. The absorption spectra of Cu-MNDTT complex versus the blank and the ligand (MNDTT) in methanol was recorded in the wavelength region of 200–800 nm (Figure 2). The ligand shows a maximum wavelength at 346 nm while the spectrum of Cu-MNDTT reveals a maximum at 475 nm which increased as a function of Cu/MNDTT molar ratio according to the curve reported in Figure 3.

**Figure 4:**
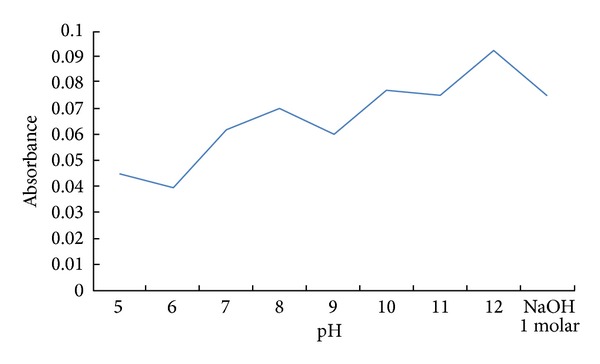
Influence of pH on the absorbance of Cu-MNDTT complex.

*Effect of pH*. The influence of pH on the complex formation using Britton-Robinson buffer (pH = 5–12) and 1 M NaOH was studied by measuring the real absorbance of the solution containing Cu(II) (20 *μ*g/mL) in the presence of the reagent MNDTT against the reagent blank. The results illustrated in Figure 4 reveal that maximum absorbance of the colored complex was obtained at pH = 12. Comparing the results obtained in the absence of buffer (Table 1) with the above values reveals that the absorbance of the produced complex decreased in the presence of aqua buffer solution. Thus, in the subsequent work, no buffer was added.

*Effect of Surfactant*. The effect of cationic (cetrimide), anionic (sodium lauryl sulfate), and nonionic (tween 80) surfactants was studied and results showed that surfactants caused turbidity in solution in different surfactant concentrations. Therefore, this method has been used in the absence of surfactants.

*Stability of Complex*. To study the stability of Cu-MNDTT complex, the absorbance of a 25 *μ*g/mL solution of Cu(II) at the optimum condition was recorded over a period of 3 h with an interval of 30 min, after 24 and 48 hours. The results showed that the complex was completely stable for at least 3 hours and there were no significant changes (<3%) in the absorbance of the complex after 24 hours.

**Figure 5:**
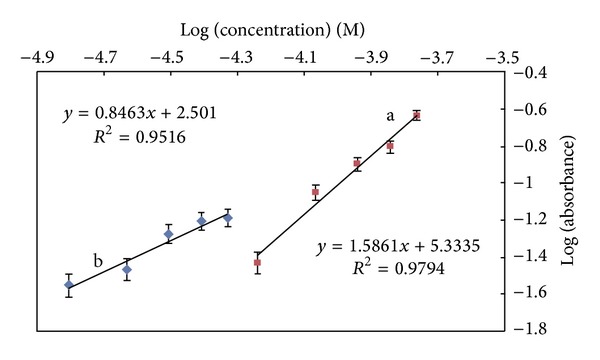
Limiting logarithmic plot for ratio of Cu(II) and reagent (MNDTT); (a) log abs versus log[MNDTT] and (b) log abs versus log [Cu].

*Stoichiometry of the Complex*. The chemical structure of Cu-MNDTT complex was determined by limiting logarithmic method. As it is shown in Figure 5 the logarithm of absorbance intensities of Cu-MNDTT complex versus log [Cu] 1.57 × 10^−5^–4.68 × 10^−5^ M) at fixed concentration of MNDTT (5.58 × 10^−5^ M) and log [MNDTT] (5.75 × 10^−5^–1.72 × 10^−4^ M) at fixed concentration of Cu(II) (1.57 × 10^−5^ M) were plotted. The values of the slopes of these lines were 0.8463 and 1.586, confirming the 1 : 2 ratios for the complex formation reaction.

## 4. Validation of the Method

*Linearity, LOD, and LOQ*. The opposed method was calibrated using 6 series in the range of 2.5–20 *μ*g/mL. The analytical parameters of the proposed method are given in Table 2. The detection limit (3 SD/*K*) and quantification limit (10 SD/*K*) (where SD is the standard deviation of the *y*-intercept and *K* represents the slope of the straight line), as defined by IUPAC, were found to be 0.33 and 1.10 *μ*g/mL, respectively [27].

*Precision and Accuracy*. The accuracy and precision of the proposed method were determined at three different concentrations within the same day (*n* = 3) and over three different days (*n* = 9). Percentage relative standard deviation (RSD%) as precision and percentage relative error (Er%), which shows accuracy of the suggested method, was less than 3.68% and 2.40%, respectively. This indicates good accuracy and precision of the method (Table 3).

*Study of Interferences*. The effect of diverse ions was determined using a standard solution containing 20 *μ*g/mL of Cu(II) and 20 *μ*g/mL of the studied ions. The method was completed according to the general procedure and the absorbance value was obtained against the reagent blank at 475 nm. Similar to the results obtained for the naphthyl derivative of 1,2,4-triasine-3-thione (6-(2-naphthyl)-2,3-dihydro-1,2,4-triasine-3-thione) obtained in previous study, the new reagent had no interference with Fe(II), Ba(II), Mg(II), Ca(II), Cd(II), Co(II), Mn(II), and Sr(II). Ni(II), Hg(II), and Pd(II) ions form complexes with MNDTT which have maximum absorbance between 400 and 500 nm [17]. As there was no suitable masking agent for these ions, derivative spectrophotometric method has been recommended for analyzing Cu(II) in the presence of Ni, Hg, or Pd ions.

*Application of the Method to Real Sample*. In order to evaluate the analytical applicability of the proposed method, it was applied for the determination of Cu(II) in a dental amalgam 110-plus. The proposed method was compared with the atomic absorption method. The results and recoveries presented in Table 4 indicate the percentage of recovery 97.5 and 96.5 for spectrophotometry and atomic absorption method, respectively. Using the two-tailed *t*-test and *f*-test methods, it was revealed that there was no significant difference between the results obtained from these two methods (*P* value > 0.05).

*Relative Recovery*. The relative recovery was determined using the standard addition method (*n* = 3). The percent relative recovery of 101.66 ± 1.24 indicates that no interference with other components in amalgam has been observed.

## 5. Conclusion

A simple, rapid, sensitive, and accurate method for determination of Cu(II) using the newly synthesized reagent, 6-(2-methoxynaphthyl)-2,3-dihydro-1,2,4-triazine-3-thion, was developed. The proposed method gave a low LOD and a good RSD. Comparison of characteristic features of some spectrophotometric methods reported earlier for the determination of copper reveals the suitability of the present work in terms of molar absorptivity, linear range, interferences, LOD, and so forth (Table 5). The use of MNDTT as a complexing reagent was utilized for nonextractive determination of Cu(II) in dental amalgam. The results were in good agreement with the atomic absorption method. Additionally, the method was much safer, since only a small amount of methanol was used which is considered as “green chemistry” for determination process. Therefore, the proposed method can be recommended for pharmaceutical and industrial samples.

## Figures and Tables

**Table 1:** Effect of the solvent system on the absorbance value of Cu(II)-MNDTT complex (20 µg Cu(II)/mL).

Solvent	0.1 M NaOH	Acetonitrile	Methanol	Ethanol
*λ*_max⁡_	476	482	475	479
Absorbance	0.05	0.108	0176	0143

**Table 2:** Analytical parameters of calibration curves of copper (II)-reagent (*n* = 9).

Parameters	
Linearity	2.50–20.00 µg/mL
Limit of detection	0.33 µg/mL
Limit of quantification	1.10 µg/mL
Molar absorptivity	4350.5
Regression equation	*Y* = 0.01286*x* + 0.01213
SD of slope	8.66 × 10^−5^
RSD of slope	0.57
SD of intercept	3.60 × 10^−4^
Coefficient correlation	0.992

**Table 3:** Accuracy and precision data for determination of copper in one day (*n* = 3) and three subsequent days (*n* = 9).

Added (µg/mL)	Found (µg/mL)	CV%	Error%
Within-day (*n* = 3)			
5.00	4.88 ± 0.14	2.87	−2.40
10.00	10.14 ± 0.13	1.28	1.40
17.50	17.43 ± 0.06	0.34	−0.40
Between-day (*n* = 9)			
5.00	4.89 ± 0.18	3.68	−2.20
10.00	10.17 ± 0.13	1.28	1.70
17.50	17.36 ± 0.11	0.63	−0.80

**Table 4:** Application of the proposed method to the determination of Cu(II) in amalgam (*n* = 3).

Compound	Label claimed (mg)	Found (mean ± sd)		Statistical tests*	
		Proposed method	A. A. method		
110-plus	10.00	9.75 ± 0.07	9.66 ± 0.10	*t* = 0.265	*F* = 0.647

*Theoretical values of *t* and *F* at *P* = 0.05 and 95% confidence are 2.776 and 6.388, respectively.

**Table 5:** Comparison of reagents for the spectrophotometric determination of copper (II).

Reagent	Molar absorptivity	*λ*_max⁡_ (nm)	Linear range	Extractant	LOD	Reference
Chloro-(phenyl)glyoxime	8 × 10^3^	290.5	0.1–10 (µg/mL)	—	0.01 (µg/mL)	[21]
1-Phenyl-1,2-propanedione-2-oxime thiosemicarbazone	5.5 × 10^3^	465	0.35–7.63 (µg/mL)	—	—	[22]
NDTT	—	501	1–30 (µg/mL)	Chloroform	0.26 (µg/mL)	[18]
meso-HMPAO	338	497	0.5–370 (µg/mL)	—	0.50 (µg/mL)	[23]
HMBO	7 × 10^2^	400	0–31.75 (µg/10 mL)	Chloroform	—	[24]
MNDTT	4.4 × 10^3^	475	2.5–20 (µg/10 mL)	—	0.33 (µg/10 mL)	Present method
